# Green, Safe, Durable, Printed Fabric Hygroelectric Generators for Wearable Systems

**DOI:** 10.1002/adma.202502091

**Published:** 2025-05-20

**Authors:** Renbo Zhu, Tongyao Liu, Andrew Balilonda, Yonghui Luo, Kitming Ma, Xiaoming Tao

**Affiliations:** ^1^ Research Institute for Intelligent Wearable Systems The Hong Kong Polytechnic University Hong Kong 999077 China; ^2^ School of Fashion and Textiles The Hong Kong Polytechnic University Hong Kong 999077 China

**Keywords:** green materials, hydrogel, moisture, water absorption, wearable electronics

## Abstract

Hygroelectric generators, converting energy from moisture into electricity, have attracted great interest due to sustainable and ubiquitous moisture in the environment. However, it is absolutely necessary to replace the fragile and noxious materials reported previously in the hygroelectric generators before real applications for wearables. Herein, a green hygroelectric generator with a high current density is designed for the first time by printing functional materials that are abundant, safe to humans and environments. By engineering printable hydrogel through the synergistic effect of water absorption and ion migration on the fabric, the wearable fabric hygroelectric generators deliver a high open‐circuit voltage of 1.2 V with a remarkable short‐circuit current density of 1.0 mA·cm^−2^, more than 7 times that of most reported hygroelectric generators. The devices show no performance declination after long‐term storage and bending tests due to the design of stable hydrogel and robust electrode/hydrogel interfaces. Moreover, the devices with cross‐finger structures achieve a facile scalable integration for enhanced electric outputs. Exemplifying applications illustrate the great potential of the printed fabric hygroelectric generators as a direct current power supply for wearable applications. This work sheds light on a novel avenue to design safe and environmentally friendly energy harvesting devices for practical applications.

## Graphical abstract

This work achieves a green hygroelectric generator with enhanced electric outputs. All chemicals and materials used in the hygroelectric generators are safe for human and environmentally friendly. The device achieves an open‐circuit voltage of 1.2 V and a short‐circuit current of 1.0 mA·cm^−2^ at room humidity of 55% through the synergistic effect of ion migration and water absorption.

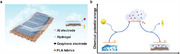

## 1 Introduction

With the development of wearable electronic devices, such as health monitoring devices and intelligent interactive systems, the demand of continuous power supply is increasing to guarantee the stable operation of wearable devices.^[^ ^1^ ^]^ However, the power supply system based on an ionic battery delivers a limited capacity and needs to be frequently charged or replaced, which leads to inconsistent and unsatisfactory operation of wearable devices.^[^ ^2^ ^]^ While traditional energy harvesting technologies for power supply devices (e.g., solar cells or piezoelectric generators) are incapable of providing continuous power supply due to limited operation scenarios (e.g., light or movement).^[^ ^3^ ^]^ Recently, hygroelectric generator, absorbing moisture from the environment and converting chemical potential energy into electricity (Figure S1, Supporting Information), has attracted great attention in continuous electricity generation due to abundant and ubiquitous moisture in the atmosphere.^[^ ^4^ ^]^ Thus, hygroelectric generator is a promising candidate in achieving continuous power supply for wearable electronics in a wide range of environments.

Generally, hygroelectric generator generates electricity by absorbing moisture with hydrophilic layer and forming a water gradient along the asymmetrical device structure, which contributes to ion migration and charge separation.^[^ ^5^ ^]^ The electric outputs (<1 V, several microamperes) of hygroelectric generators are still insufficient to power a great number of wearable electronic devices.^[^ ^6^ ^]^ To enhance the electric outputs, great efforts have been made to improve the water absorption and ionic conductivity of hydrophilic layer.^[^ ^7^ ^]^ However, the materials (e.g., LiCl or acids) used in the functional layer is harmful to the human body or environment,^[^ ^8^ ^]^ which impedes its development and practical application in wearable electronic systems. Sodium polyacrylate (PAAS), as super‐absorbent polymer (SAP), can absorb several hundred times as much water as its mass.^[^ ^9^ ^]^ Besides, PAAS is non‐toxic and environmentally friendly,^[^ ^10^ ^]^ making it a good potential hydrophilic material in designing functional layers of hygroelectric generators.

**Figure 1:**
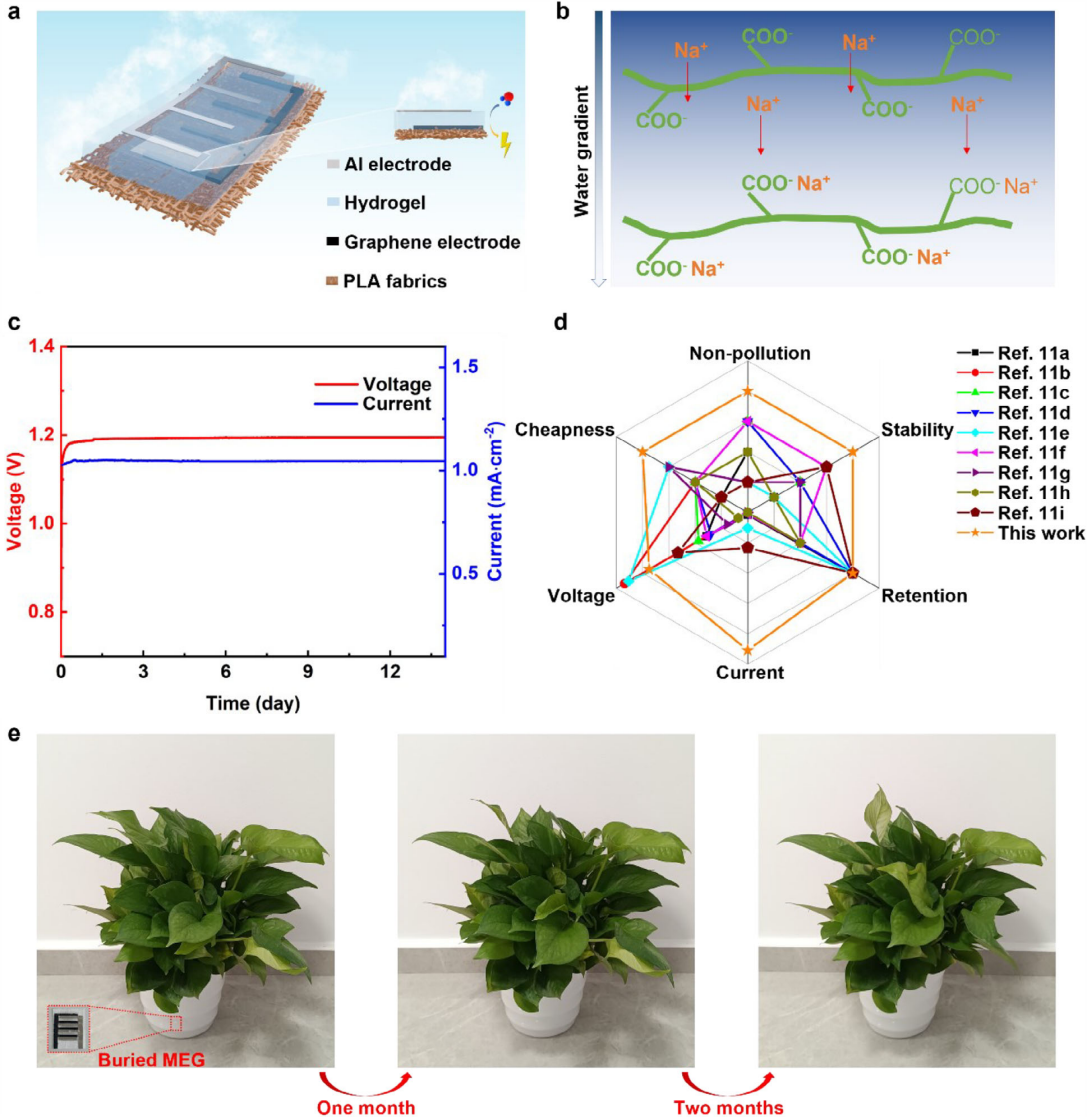
Design and performance of hygroelectric generators. a) Schematical illustration of hygroelectric generator with cross‐finger structures. b) Mechanism illustration of ion migration and electricity generation under water gradient. c) The open‐circuit voltage and short‐circuit current of hygroelectric generator at room humidity of 55% and room temperature of 25 °C over 2 weeks. d) The performance comparison of hygroelectric generators in this work with the other reported hygroelectric generators.^[^ ^11^ ^]^ e) The growth of green plants with hygroelectric generator units buried in the soils for three months.

In this work, we propose a green hygroelectric generator with high electric outputs for the first time (**Figure** **1**a), based on PAAS/sodium chloride (NaCl) with optimized moisture absorption and high ionic conductivity. All chemicals and materials used in the hydroelectric generators are safe to humans and environmentally friendly. The single hygroelectric generator unit delivered a high open‐circuit voltage of 1.2 V and a remarkable short‐circuit current of 1.0 mA·cm^−2^ at room humidity of 55%. The mechanisms of enhanced outputs are well elaborated through the synergistic effect of ion migration and water absorption. Besides, we design a facile printing approach to scale up hygroelectric generator units with cross‐finger structures to enhance its electric outputs, which ensures the stable and continuous operation of commercial electronic devices. This work solves a series of critical challenges of practical application with hygroelectric generator devices as power supply and contributes to a more reliable self‐powered wearable electronic system.

## 2 Results and Discussion

### 2.1 Design of Green Hygroelectric Generator

A wearable and green hygroelectric generator was designed by using poly(lactic acid) (PLA) fabric as substrate, followed by printing graphene as bottom electrode, hot pressing of graphene/PLA, printing PAAS/NaCl paste as a functional layer, and attaching aluminium (Al) as top electrode (Figure S2, Supporting Information). The PAAS was selected as the skeleton for water absorption and ion migration. NaCl was added to improve the content of charge carriers for better ionic conductivity and higher current outputs. Due to the asymmetrical moisture absorption, the water gradient forms along the thickness of PAAS/NaCl gel (Figure 1b). The higher water content in the upper area exposed to the environmental moisture contributes to the more mobilized Na^+^ ions and a gradient of mobilized ions, which drives Na^+^ ion migration along the water gradient. Compared with Na^+^ ions, the anions (–COO^−^) are more immobilized as anions are bonded to polymer chains with higher molecular weight by covalent bond, which leads to different ion migration rates for charge separation and electricity generation. The single hygroelectric generator unit with a size of 1.0 cm^2^ delivered a sustainable open‐circuit voltage of 1.2 V over 2 weeks at room humidity of 55% (Figure 1c; Figure S3, Supporting Information). Besides, the hygroelectric generator unit achieved a significant current output of 1.0 mA·cm^−2^, more than 7 times that of most reported hygroelectric generator devices (Table S1, Supporting Information). Moreover, the hygroelectric generator devices exhibit a great comprehensive performance in regard of non‐pollution, great stability, good retention, high voltage/current output and low cost (Figure 1d), compared with other reported hygroelectric generator.^[^ ^11^ ^]^ The hygroelectric generator units buried in the soils do not threat the growth of green plants (Figure 1e), demonstrating that all materials used in the hygroelectric generator units are environmentally friendly in the green aspect (Table S2, Supporting Information) and are safe as wearable application. Thus, hygroelectric generator devices in this work are quite promising as power supply in achieving practical application of wearable electronic device.

### 2.2 Electrical Performance of Green Hygroelectric Generator

**Figure 2:**
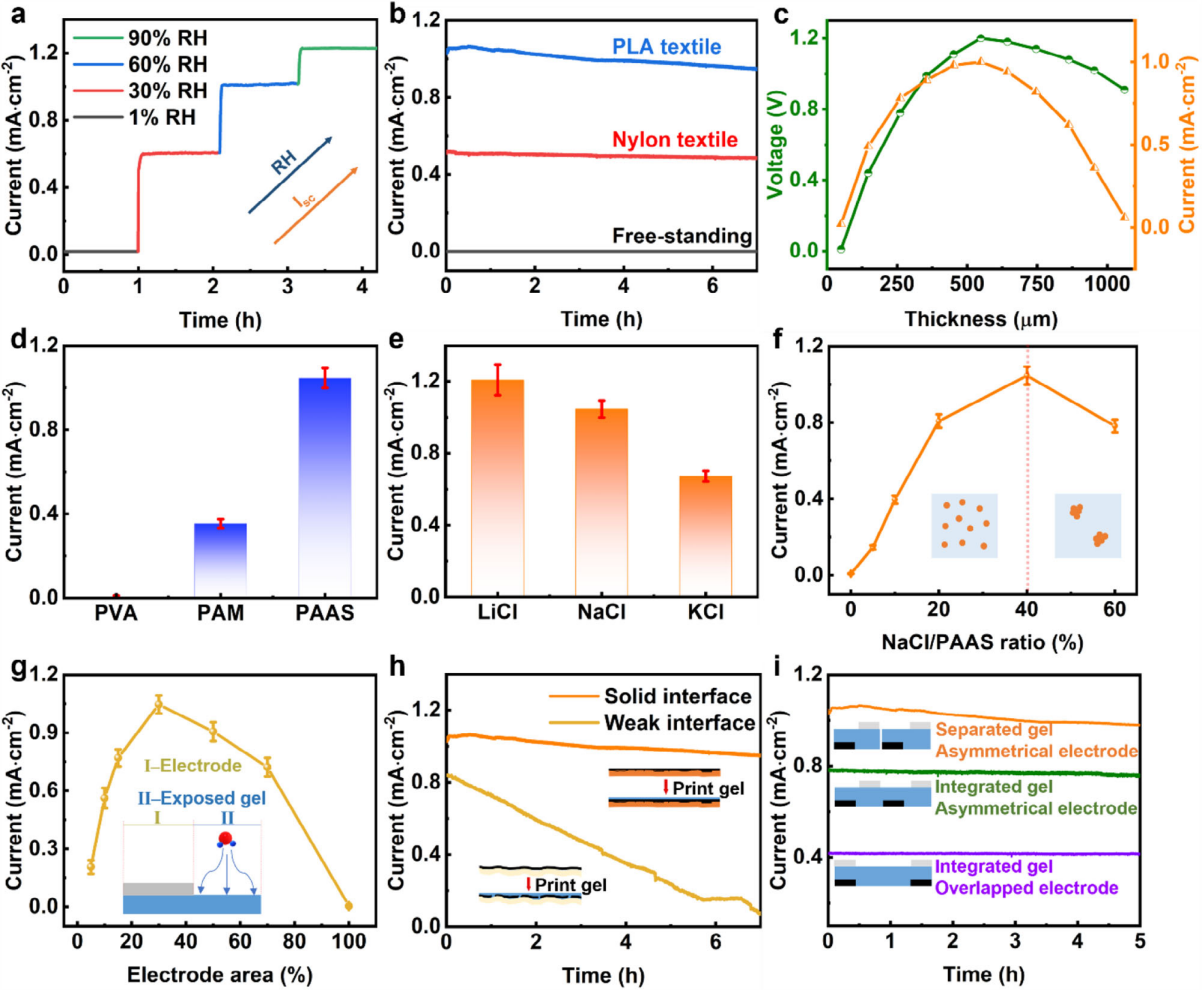
Electric output of green hygroelectric generator. a) Current density of hygroelectric generator at different RH. b) Current density of hygroelectric generator fabricated on different fabrics as substrate. c) Open‐circuit voltage and short‐circuit current of hygroelectric generator with different thickness of functional layer. d) Current density of hygroelectric generator with different polymers as skeleton in the functional layer. e) Current density of hygroelectric generator with LiCl, NaCl or KCl embedded in PAAS as functional layer. f) Current density of hygroelectric generator with different ratios of NaCl and PAAS. g) Current density of hygroelectric generator printed with different areas of top electrode. h) Current density of hygroelectric generator with and without hot pressing of graphene electrode on the PLA fabric. i) Current density of hygroelectric generator with different printed structures.

The electrical outputs of hygroelectric generator are closely related to the synergistic effect of ion migration and water absorption under water gradient. To address the effect of water gradient on the electrical outputs, the single hygroelectric generator unit with different water gradients was investigated. Once exposed to an environment with higher relative humidity (RH), the hygroelectric generator unit exhibits a greater water gradient along the thickness and delivers a higher electric output. As shown in **Figure** **2**a, the current output of the hygroelectric generator is 0.02, 0.60, 1.00, 1.23 mA·cm^−2^ with an RH of 1%, 30%, 60%, 90%, respectively. The environment with a higher RH contributes to a greater driving force of ion migration under a higher water gradient. Besides, the water gradient is constructed by asymmetrical moisture absorption and varies on the various fabric substrates with different moisture permeability. The moisture permeability of PLA fabrics is lower than that of Nylon fabrics (Figure S4, Supporting Information). Thus, the hygroelectric generator on PLA fabric delivers a higher water gradient and current output (Figure 2b). The hygroelectric generators on PLA fabric, Nylon fabric, no fabric (free‐standing) delivered a current output of 1.00 mA·cm^−2^, 0.51 mA·cm^−2^, 0 mA·cm^−2^, respectively, at room humidity of 55%. The free‐standing hygroelectric generators without fabrics as substrate are exposed to moisture symmetrically without water gradient and show no obvious electric output, which also demonstrates that water gradient is the driving force in the electricity generation of hygroelectric generator. By dropping water on the exposed surface of hygroelectric generator, the current increases with the greater water gradient and then drops with the decreased water gradient (Figure S5, Supporting Information). Moreover, the voltage and current increase with gel thickness when gel thickness is lower than 500 µm (Figure 2c), which is attributed to a higher water gradient in a thicker gel. The voltage and current decrease with gel thickness when gel is thicker than 500 µm as the thicker film exhibits a longer migration path between the top and bottom side.^[^ ^12^ ^]^ Therefore, the film thickness is optimized by balancing water gradient and ionic resistance. The hygroelectric generator exhibits a good operation stability in the cycles of varied RH (Figure S6, Supporting Information).

The higher ionic conductivity of functional layer is critical in achieving a higher current output, which is investigated by optimizing the composite of functional layer in this work. Various polymers (Figure 2d) as skeleton of functional layer in the hygroelectric generator were fabricated and contributed to a current output of 0.01 mA·cm^−2^ (PVA), 0.35 mA·cm^−2^ (PAM), 1.00 mA·cm^−2^ (PAAS) at room humidity of 55%. The functional layers with different polymers exhibit various water contents by different hydrophilic groups (e.g., –OH in PVA, –NH_2_ in PAM, –COONa in PAAS). PAAS gel shows a high content of super hydrophilic –COONa and endows the super‐absorbent functional layer with high water contents (Figure S7, Supporting Information), which leads to a higher ionic conductivity (Figure S8, Supporting Information) and current outputs. Besides, the hygroelectric generators with lithium chloride (LiCl), NaCl, potassium chloride (KCl) delivered a current output of 1.21, 1.00, 0.67 mA·cm^−2^, respectively (Figure 2e). The ionic radius of Li^+^, Na^+^, and K^+^ is 0.76 Å, 1.02 Å, 1.38 Å, respectively, which leads to different ion migration rates (Li^+^ > Na^+^ > K^+^).^[^ ^13^ ^]^ Thus, the functional layer with LiCl shows a higher ionic conductivity and current output. However, LiCl is not suitable for practical application as it is toxic to the human body and environment.^[^ ^14^ ^]^ Thus, the functional layer with NaCl is a better candidate in achieving a high ionic conductivity as well as an environmentally friendly application. By increasing NaCl ratio in the functional layer (Figure 2f), the current output increases and then decreases when the NaCl/PAAS ratio is higher than 40%. The inorganic salts embedded in the polymers improve ionic conductivity by enriching charge carriers. However, the excess NaCl salts added into PAAS cause salt integration and lower ionic conductivity, resulting in the decreased current outputs. The ion migration in the functional layer is also enhanced by increasing the operation temperatures for higher current outputs (Figure S9, Supporting Information).

Moreover, the device structure was modified to achieve optimized electric outputs with better water absorption and charge migration. The area of top side is consisted of the electrode area and exposed area, which works as current collection and water absorption, respectively. The current output increases with the ratio of electrode area and exposed area as more electrode areas get involved in the current collection. When the electrode area is higher than 30%, the current output decreases with electrode area due to insufficient water absorption by decreased exposed areas (Figure 2g). Thus, the electrode area of hygroelectric generator will be set to 30% to achieve highest current density of 1.00 mA·cm^−2^ by balancing current collection and water absorption. While the voltages of hygroelectric generator show no obvious change with different electrode areas as the voltage is determined by the vertical ion gradient (Figure 1b; Figure S10, Supporting Information). The cyclic voltammetry curve of hygroelectric generator with graphene as bottom electrode, PAAS/NaCl as functional layer, Al as top electrode, exhibits no reaction peak (Figure S11, Supporting Information), while no Al^3+^ is detected in the PAAS/NaCl layer after operation with Al electrodes (Figure S12, Supporting Information), demonstrating no chemical reaction occurs for electricity generation. The hygroelectric generators with different top electrodes also deliver similar electric outputs at room humidity of 55% (Figure S13, Supporting Information). Besides, the output retention of current is improved by hot pressing of graphene electrode on PLA fabric after printing graphene electrode (Figure 2h; Figure S2, Supporting Information). The more stable current output is attributed to the embedded structure of the graphene electrode and PLA fabric after hot pressing (Figure S14, Supporting Information), which exhibits a stable interface and a low resistance. The conductivity of the graphene electrode on PLA improves from 8 kΩ·cm^−1^ to 46 Ω·cm^−1^ after hot pressing (Figure S15, Supporting Information). The interface of the graphene electrode and PLA fabric without hot pressing is weak with a higher resistance after printing the functional layer. Moreover, the different structures of hygroelectric generator units (Figure 2i) are designed to regulate electric outputs of multiple integrated units. It is found that the separated functional layers with cross‐finger electrodes lead to higher electric outputs due to the uniform current collection of multiple hygroelectric generator units.

### 2.3 Mechanism of Electricity Generation

**Figure 3:**
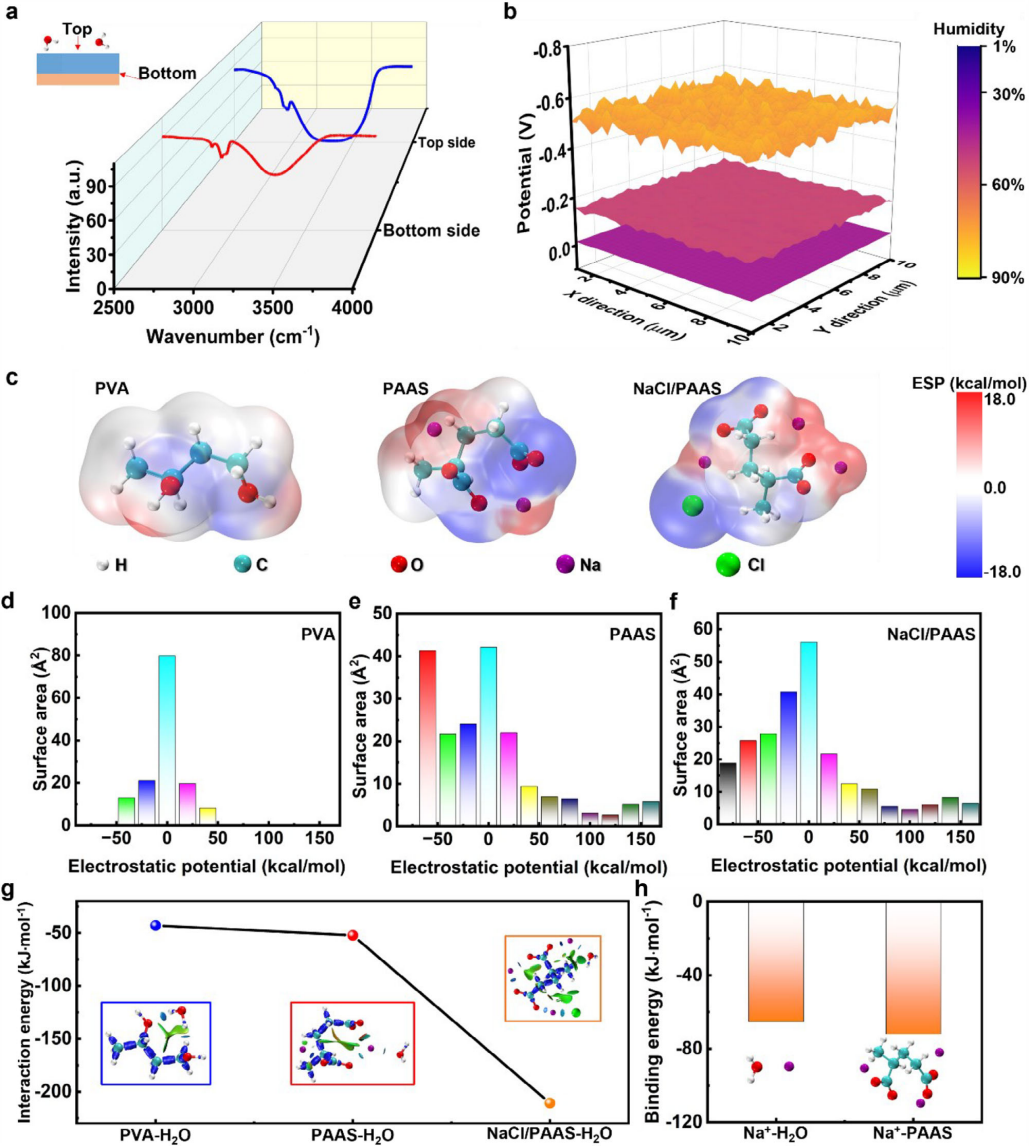
Mechanism illustration of electricity generation of hygroelectric generators. a) FTIR of top and bottom side of PAAS/NaCl gel after exposing gel to 55% RH for 2 weeks. The gel was peeled off from the fabric before recording FTIR of bottom side. b) Potential changes of top side of PAAS/NaCl gel at different RH of 1%, 30%, 60% from KPFM tests after exposing gel to a controlled RH for 2 weeks. c) The ESP distribution of PVA, PAAS and PAAS/NaCl from left to right by DFT calculations. d,e,f) Surface area and corresponding area percent in each ESP range on the vdW surface of PVA, PAAS and PAAS/NaCl, respectively. g) Interaction energies of PVA‐H_2_O, PAAS‐H_2_O and PAAS/NaCl‐H_2_O from IRI maps. h) Binding energy and specific adsorption models of Na^+^‐H_2_O and Na^+^‐PAAS in the gas phase.

The charge separation and electricity generation are driven by water gradient, which is further investigated with water and ion diffusion process by various characterization methods and density functional theory (DFT) calculations. By exposing printed PAAS/NaCl gel on PLA fabric to 55% RH for 2 weeks, Fourier Transform Infrared (FTIR) Spectroscopy of the top side was recorded after operation for 2 weeks, followed by testing bottom side with FTIR by peeling PAAS/NaCl gel off from PLA fabric. The strength of ν(–OH) at ∼3290 cm^−1^ of top side is higher than that of bottom (**Figure** **3**a), which indicates that PAAS/NaCl gel shows a higher water content on the top side, indicating that a vertical water gradient was constructed accordingly even after operation for 2 weeks.^[^ ^15^ ^]^ To demonstrate charge separation under water gradient, the Kelvin probe force microscope (KPFM) was adopted to check the surface charge variation under different RH.^[^ ^16^ ^]^ The KPFM shows that no obvious surface potential is found at 1% RH, suggesting that almost no charge separation occurred without water gradient. As RH increases, the surface potential becomes more negative (−0.02 V at 1% RH, −0.16 V at 30% RH, −0.54 V at 60% RH in Figure 2b) as more mobilized Na^+^ ions migrate from top side to bottom side at a higher RH, leaving the top side with more negative charges (e.g., –COO^−^), revealing charge separation and ion migration by water gradient.

The improved water absorption of the designed PAAS/NaCl gel is investigated by electrostatic potential (ESP) distribution on the molecular van der Waals (vdW) surface from DFT calculations. The ESP maps of PAAS, PAAS/NaCl reveal broader distributions with more positive (red) and negative (blue) regions than that of PVA (Figure 3c; Figure S16, Supporting Information), which contributes to the formation of electrostatic gradient and drives ions migration along polymer skeleton for charge separation.^[^ ^17^ ^]^ In addition, the minimum/maximum electrostatic potential of PVA, PAAS, PAAS/NaCl is −40/40, −60/160, −80/160 kcal·mol^−1^, respectively (Figure 3d–f; Figure S17, Supporting Information). The higher absolute electrostatic potential indicates better water absorption of polymers, which is attributed to that PAAS with more abundant functional groups (e.g., –COONa) captures moisture better than PVA for greater electricity generation. While the slightly higher ratio of absolute electrostatic potential value of polymers (Figure 3e,f) after adding NaCl into PAAS also indicates that the addition of NaCl leads to a slight increase of moisture absorption by NaCl hydration. ^[^ ^17^ ^]^

Interaction region indicator (IRI) by DFT calculations is critical in illustrating the interaction between polymer and H_2_O (Figure S18, Supporting Information). The calculated absorption energy of PVA, PAAS, PAAS/NaCl is −42.9, −52.3, −210.8 kJ·mol^−1^, respectively (Figure 3g). The high absolute value of absorption energy by PAAS/NaCl reveals the strong attraction between H_2_O and polymers for enhanced moisture absorption and electric outputs. The structure of PAAS/NaCl molecules exhibits a more intensive interaction with H_2_O in the scatter maps between IRI and sign(λ_2_)ρ of PAAS/NaCl‐H_2_O (Figure S19, Supporting Information), which is attributed to the greater moisture absorption and more dissociated Na^+^ ions for charge separation. In addition, the binding energy of Na^+^‐PAAS (−74.0 kJ·mol^−1^) is more negative than that of Na^+^‐H_2_O (−65.2 kJ·mol^−1^), suggesting that mobilized Na^+^ ions are more likely to move along polymer chains instead of water molecules.^[^ ^17^ ^]^ Thus, PAAS as a skeleton not only absorbs moisture by the great hydrophilicity but also works as migration paths of charge carriers through an elaborated kinetic process.

### 2.4 Unit Integration for Enhanced Electric Outputs

**Figure 4:**
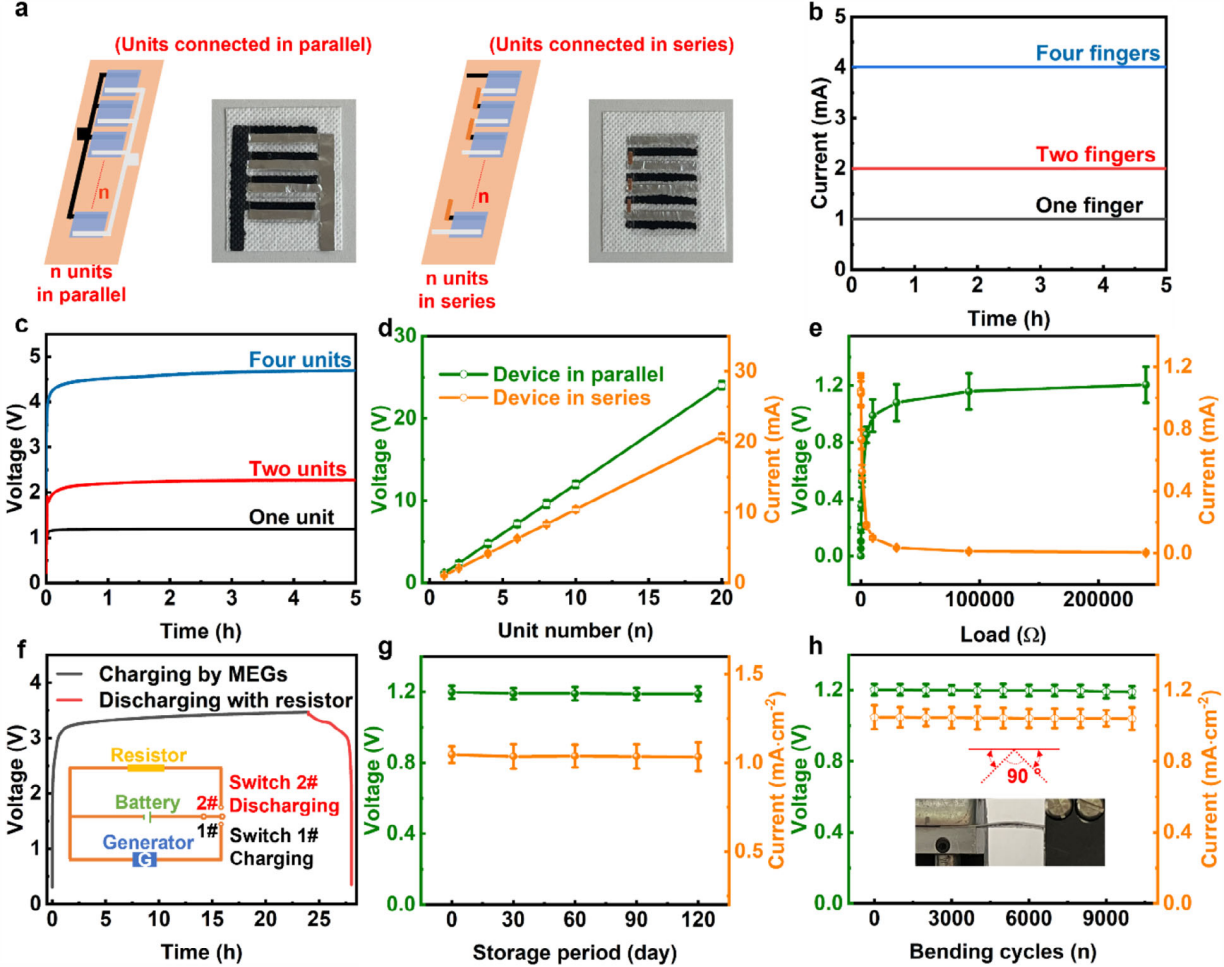
Integrated hygroelectric generator units as wearable power supply as 55% RH. a) Schematical illustration and photo of hygroelectric generator units connected in series or parallel. b) Short‐circuit current of hygroelectric generator with different units connected in parallel. c) Open‐circuit voltage of hygroelectric generator with different units connected in series. d) Electric output of hygroelectric generator with different units connected in series or parallel. e) Electric outputs of external resistors connected to a single hygroelectric generator unit. f) The voltage outputs of a commercial Li‐battery. The battery was charged by 3 hygroelectric generator units connected in series and was discharged with an external resistor. g) The electric outputs of a single hygroelectric generator unit with different storage periods at 25 °C and 55% RH. h) The electric outputs of a single hygroelectric generator unit with different bending cycles and a bending angle of 90°.

To power practical electronic devices demanding high electric power, the printable cross‐finger structures are designed in this work and achieve a facile integration of multiple units for enhanced output. As shown in **Figure** **4**a, the hygroelectric generator units were connected in parallel by printing multiple finger‐structure graphene electrodes onto PLA fabric as bottom electrodes, followed by printing separated PAAS/NaCl gels as functional layers and attaching finger‐structure Al electrodes as top electrodes. For hygroelectric generator units connected in series, the top electrode of a hygroelectric generator unit was connected to the bottom electrode of next hygroelectric generator unit by Cu tape. The hygroelectric generator devices connected in parallel delivered sustainable current outputs of 1.0, 2.1, 4.2 mA (Figure 4b) with single unit, 2 units, 4 units, respectively. While voltages of hygroelectric generator units connected in series were 1.2 V, 2.4 V, 4.8 V (Figure 4c) with single unit, 2 units, 4 units, respectively. The voltage and current outputs show a linear increase with unit number, suggesting no obvious power loss occurs in this facile unit integration. The voltage and current of hygroelectric generators with 20 units are 24.0 V and 20.8 mA (Figure 4d), respectively, which are sufficient in powering various practical electronic devices. This printing design is inspiring in the fast scalable integration of hygroelectric generator units with high electric outputs and low costs.

As power supply device, the energy harvested by hygroelectric generator devices can be used to power electronic devices directly. The electric output of external electronic devices with different resistances is investigate by connecting hygroelectric generators with different resistors, as shown in Figure 4e. With the increase of external resistance, the external resistors show increased voltage outputs and decreased current outputs (Figure 4e), which achieve a highest power output of 0.27 mW·cm^−2^ at 55% RH (Figure S20, Supporting Information). Besides, the electricity generated by hygroelectric generator is stored in the Li‐battery charged by 3 hygroelectric generator units connected in series (Figure 4f), which extends operation life of Li‐battery by extra 25%. In the charging circuit (Figure S21a, Supporting Information), the current of the battery and external resistor is 0.74 and 0.01 mA, respectively, with a load of 90 kΩ. The current of the battery and external resistor is 2 mA and 0.04 mA, respectively, with a load of 180 Ω, which achieves a mode switch between charging Li‐battery and powering electronic devices by adjusting the resistance of external load. The different commercial capacitors charged by one device unit are also demonstrated to store energy from hygroelectric generator successfully (Figure S22, Supporting Information).

The long‐term operation and wearability are important in evaluating hygroelectric generator devices as wearable power supply. The hygroelectric generator unit retained 99.2% maximum voltage output (V_max_) and 99.8% maximum current output (I_max_) with no obvious performance decline after storage at 25 °C and 55% RH for 6 months (Figure 4g). In addition, the hygroelectric generator was bended with a bending angle of 90° repeatedly for 10 000 times and retained 99.0% V_max_ and 99.2% I_max_ (Figure 4h; Figure S23, Supporting Information). Thus, the printed hygroelectric generators show a great potential in wearable power supply for practical application.

### 2.5 Practical Application of Green Hygroelectric Generators

**Figure 5:**
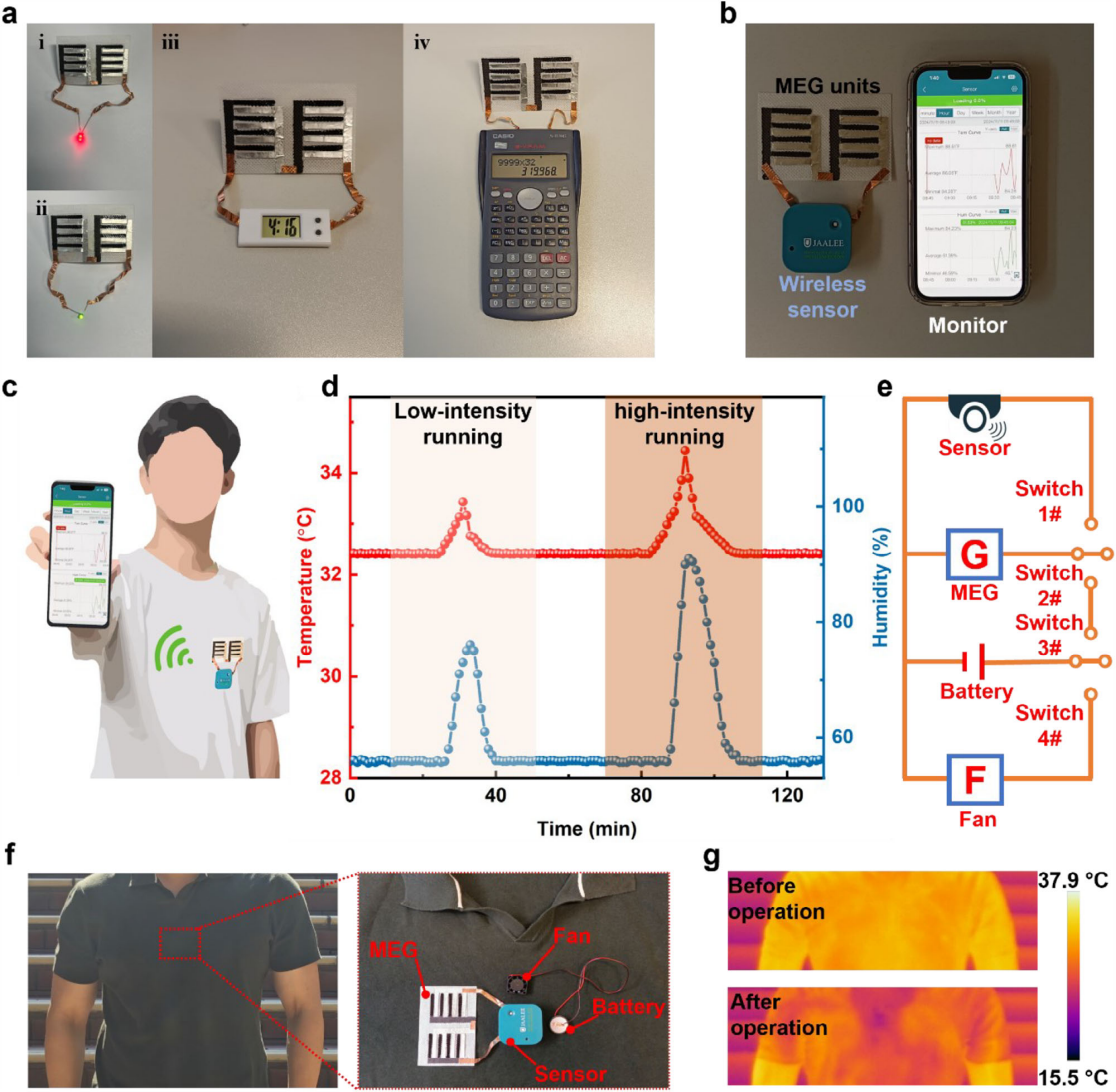
Demonstration of practical application powered by green hygroelectric generators. a) The commercial electronic devices powered by hygroelectric generator units with 2 × 4 arrays at room humidity of 55%. b) Photo of a wireless temperature/humidity sensor powered by hygroelectric generator units with 2 × 4 arrays. c) Schematic illustration of wearable monitoring system in detecting body signal. The hygroelectric generator units, wireless sensor and phone provide power supply, signal generation/transmission and signal receiver, respectively. d) The temperature and humidity signals collected on the clothes in the different states of running. e) Schematic illustration of integrated cooling and sensor system. f) Photo of the integrated cooling and sensor system attached on the inner side of clothes. g) IR image of the upper body before and after operation of integrated system.

The hygroelectric generators can be easily integrated with various types of electronic devices for practical application. As shown in **Figure** **5**a, hygroelectric generator units with 2 × 4 arrays power red LED, green LED, electronic clock, commercial calculator directly at room humidity of 55%. Besides, the hygroelectric generator units are connected to the wireless sensor, transmitting signals of temperature and humidity to the phone (Figure 5b). The flexible fabric‐based hygroelectric generators with wireless sensor are attached to the clothes and collect real‐time data from human body for activity monitoring (Figure 5c). In the low‐intensity running, the temperature and humidity near human body increased from 32.41 °C/55.9% to 33.44 °C/76.0% (Figure 5d). In the high‐intensity running, the recorded signals in the monitor show that the highest temperature and humidity is 34.46 °C and 91.0%, respectively. The wireless monitoring system can be adopted to track real‐time human activity in the different exercise intensities. Moreover, the electricity generated by hygroelectric generator units can be stored in the energy storage devices (e.g., Li‐battery in Figure 4f) to release electricity with a high current output (>20 mA in Figure S20b, Supporting Information) and power practical application with a high‐power demand. As shown in Figure 5e,f, the electric fan is connected to a Li‐battery charged by hygroelectric generators and is attached to the inner side of clothes for body cooling. The function of powering sensor, charging battery, powering electric fan is performed by switch 1#, switch 2#&3#, switch 4#, respectively. After operation for 1 min, the area covered with electric fan is cooling down and exhibits a much lower temperature of 22.70 °C (Figure 5g; Figure S24, Supporting Information).

## 3 Conclusion

In summary, we proposed a green fabric‐based hygroelectric generator by designing printable hydrogel and electrode materials, which can be used and disposed without causing harm or pollution to the human or environment. At room humidity of 55%, the hygroelectric generator delivers a high open‐circuit voltage of 1.2 V. The short‐circuit current reaches a remarkable output of 1.0 mA·cm^−2^, which is more than 7 times outputs of most reported hygroelectric generators due to great water absorption and ionic conductivity. Besides, the printable hygroelectric generator units can easily achieve scalable integration with enhanced outputs (24.0 V and 20.8 mA by 20 units). The electric performance of hygroelectric generator is stable, which exhibits no obvious decline after six‐month storage and ten thousand bending cycles. Moreover, the hygroelectric generator units successfully power a variety of commercial electronic devices for practical application (e.g., activity monitoring, body cooling). The hygroelectric generators in this work shed a light in designing safe and environment friendly hygroelectric generator with high electric outputs for practical application.

## 4 Experimental Section

### Chemicals and Materials

Sodium chloride (NaCl), potassium chloride (KCl), lithium chloride (LiCl), polyvinyl alcohol (PVA, M_w_ = 89000–98000), polyacrylamide (PAM, M_n_ = 40 000), conductive graphene inks were purchased from Sigma‐Aldrich. Al foils with a thickness of 20 µm were supplied from Shenzhen Dieckmann Tech Co., Ltd. Sodium polyacrylate (PAAS, M_w_ = 4 × 10^6^–5 × 10^6^ g·mol^−1^) was purchased from Maclin.

### Preparation of Hygroelectric Generator

To fabricate printable hydrogel, different polymer dispersions were prepared by dispersing polymers into deionized water with salts at 90 °C for 1 h with stirring for 5 h. The ratio of polymer and water was determined by suitable viscosity for printing. The cross‐finger graphene electrodes with a finger size of 1 cm^2^ were fabricated on the fabrics by printing with a designed stencil, followed with solidification by drying at 50 °C for 10 mins. The functional layers of different polymers with multiple arrays were printed onto graphene electrode with a designed stencil and then was dried at 50 °C for 5 h. The cross‐finger Al electrodes with a finger size of 1 cm^2^ were attached to the functional layer (Figure S20).

### Characterization

The relative humidity was controlled by an environmental chamber. The electric outputs of hygroelectric generators and external resistor were recorded by Keithley 2400 (Tektronix, USA). The surface potentials of hygroelectric generators at different humidity were conducted by Kelvin probe force microscopy (KPFM) with Bruker Dimension Icon machine after hygroelectric generators were placed at different humidity for 12 h. Fourier‐transform infrared spectroscopy (FTIR, PerkinElmer, Spectrum 100) was used to record wetting strates of different surfaces. The elements in the different sides of functional layer were characterized by X‐ray photoelectron spectroscopy (XPS) with the Al‐Kα radiation (8.34 Å) as a power source in the ESCALAB 250Xi (Thermo Fisher) spectrometer. Electrochemical impedance spectroscopy (EIS) was carried out by Autolab PGSTAT302N in a two‐electrode system at room humidity (RH = 55%). Cyclic voltammetry (CV) curves were recorded at a scan rate of 0.5 mV·s^−1^ by electrochemical workstation (CHI 660E).

### Quantum Chemistry Calculations

The DFT calculations were performed by Gaussian 16 software. The B3LYP functional and D3BJ dispersion correction were used to analyse the structures of PVA, PAAS, PAAS/NaCl, PVA‐H_2_O, PAAS‐H_2_O, PAAS/NaCl‐H_2_O with gas phases.^[^ ^18^ ^]^ The 6–31G(d,p) basis set was used for all atoms to optimize the geometry of polymer molecules and calculate the frequencies. The single point calculations were computed at the M062x‐D3/6‐311G(d,p) level of theory.^[^ ^19^ ^]^ Interaction region indicator (IRI) with the color mapped isosurface of PVA‐H_2_O, PAAS‐H_2_O, PAAS/NaCl‐H_2_O and electrostatic potential of PVA, PAAS, PAAS/NaCl were carried out in Multiwfn and VMD packages.^[^ ^20^ ^]^

## Conflict of Interest

The authors declare no conflict of interest.

## Supporting information



Supporting Information

## Data Availability

The data that support the findings of this study are available from the corresponding author upon reasonable request.
