# Supplementary material for: Green, Safe, Durable, Printed Fabric Hygroelectric Generators for Wearable Systems
Source: Adv Mater. 2025 May 20;37(48):2502091. doi: 10.1002/adma.202502091 (PMC12676085; doi:10.1002/adma.202502091)
Supplement: Supplementary file 1 — Supporting Information [file ADMA-37-2502091-s001.docx]

**Supporting Information**

**Green, Safe, Durable, Printed Fabric Hygroelectric Generators for Wearable Systems**

*Renbo Zhu ^a,b,#^, Tongyao Liu ^a,b,#^, Andrew Balilonda ^a,b^, Yonghui Luo ^a,b^, Kitming Ma ^a,b^, and Xiaoming Tao ^a,b,^**

^a^ Research Institute for Intelligent Wearable Systems, The Hong Kong Polytechnic University, Hong Kong 999077, China.

^b^ School of Fashion and Textiles, The Hong Kong Polytechnic University, Hong Kong 999077, China.

^#^ These authors contributed equally to this work.

E-mail: xiao-ming.tao@polyu.edu.hk


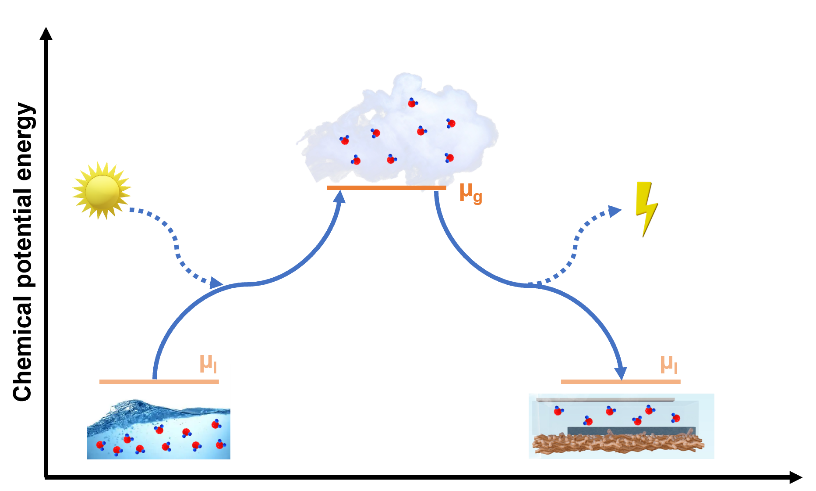


Figure S1. Schematical illustration of energy conversion between liquid water and gaseous water. The µ_l_ and µ_g_ represent chemical potential energy of liquid water and gaseous water, respectively. The water absorbs solar energy in the evaporation and then releases energy for electricity generation by water absorption with hygroelectric generators.


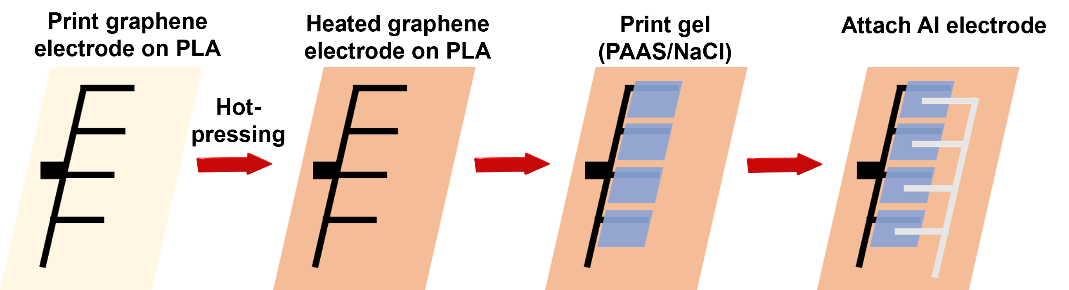


Figure S2. Fabrication illustration of fabric hygroelectric generator. The graphene electrode as bottom electrode was tightly attached to PLA fabric by hot-pressing, followed by printing functional materials with cross-finger structures and attaching Al electrode as top electrodes.


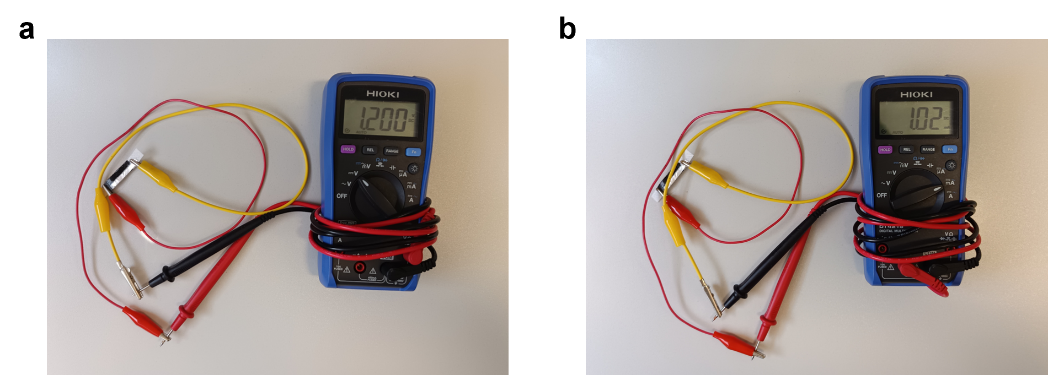


Figure S3. Photos of electric outputs of a single hygroelectric generator unit with an electrode size of 1 cm^2^ tested by a multimeter at room humidity of 55%. a) Open-circuit voltage. b) Short-circuit current.

Table S1. The summarization of recently reported hygroelectric generators

| Functional layer | RH (%) | Size (cm^2^) | Current (µA·cm^-2^) | | Voltage (V) | Output type | Hazard | Reference |
| --- | --- | --- | --- | --- | --- | --- | --- | --- |
| G.sulfurreducens Protein | 50 | 0.5 | 17 | 0.53 | | Continuous | Yes | [1] |
| rGO/GO | 80 | 3.14 | 0.038 | 1.5 | | Transient | No | [2] |
| GO/PAAS | 80 | 0.1 | 12 | 0.6 | | Continuous | No | [3] |
| Asymmetric GO | 25 | 0.04 | 0.9 | 0.45 | | Continuous | No | [4] |
| Whey protein | 40 | 0.64 | 113 | 1.45 | | Continuous | Yes | [5] |
| TiO_2_ | 85 | 1.44 | 8 | 0.52 | | Transient | Yes | [6] |
| Paper | 70 | 15 | 0.01 | 0.25 | | Transient | Yes | [7] |
| Nanofibrils | 99 | 4 | 0.011 | 0.12 | | Transient | No | [8] |
| P(VDF-TrFE)/PAN/CNW | 40 | 2 | 128 | 0.85 | | Continuous | Yes | [9] |
| H-PSS membrane | 90 | 0.25 | 120 | 0.8 | | Continuous | Yes | [10] |
| g-O-BP aerogel | 80 | 4 | 0.16 | 0.25 | | Continuous | No | [11] |
| PGA-CA hydrogel | 90 | 10800 | 130 | 0.55 | | Continuous | No | [12] |
| PDDA/NaAlg fiber | 20 | - | 1050 | 0.8 | | Continuous | Yes | [13] |
| (GO)PANI/F-Nafion (PDDA) | 94 | 1 | 8 | 0.9 | | Continuous | Yes | [14] |
| PSS/GO/GI/PVA | 15 | 1 | 7.08 | 0.55 | | Continuous | Yes | [15] |
| PAAS/NaCl | 55 | 1 | 1000 | 1.2 | | Continuous | No | This work |

Table S2. The summarization of green materials used in the hygroelectric generator

|  | PLA | Graphene | PAAS/NaCl (H_2_O) | Al |
| --- | --- | --- | --- | --- |
| Weight ratio (wt.%) | 26.5% | 0.6% | 1.9% (69.8%) | 1.2% |
| Biodegradable | Yes | Yes | No | No |
| Disposable | Yes | Yes | Yes | Yes |
| Non-toxic | Yes | Yes | Yes | Yes |
| Environmentally friendly | Yes | Yes | Yes | Yes |


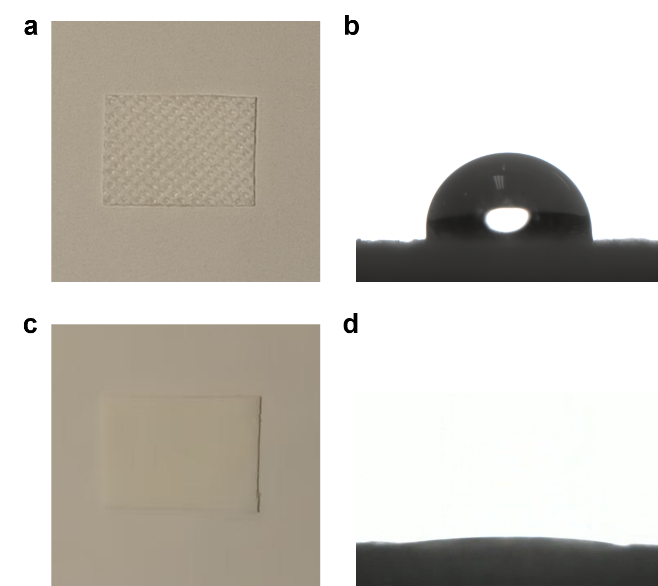


Figure S4. PLA and nylon fabrics. a) Photo of PLA fabric. b) Water contact angle of PLA fabric. c) Photo of nylon fabric. d) Water contact angle of nylon fabric. The water contact angle was recorded after dropping water for 5 mins.


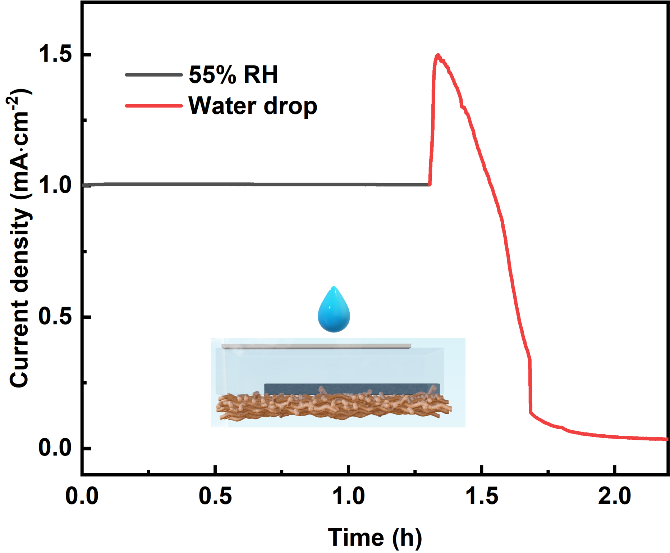


Figure S5. The current density of hygroelectric generator with 55% RH and water drop. The hygroelectric generator was placed at 55% RH and then was dropped with 0.05 g droplet of D.I. water on the top surface.


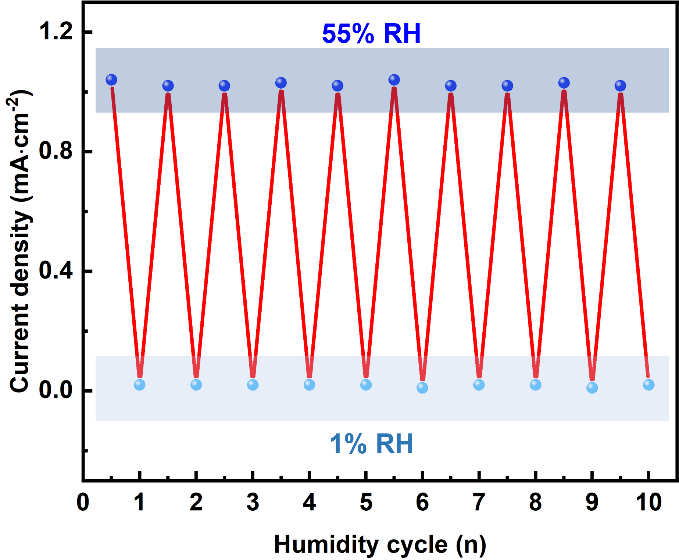


Figure S6. The current density of the hygroelectric generator unit under humidity variation cycle. The hygroelectric generator unit was placed at 55% RH for 0.5 h and then was placed at 1% RH for 0.5 h for one cycle.


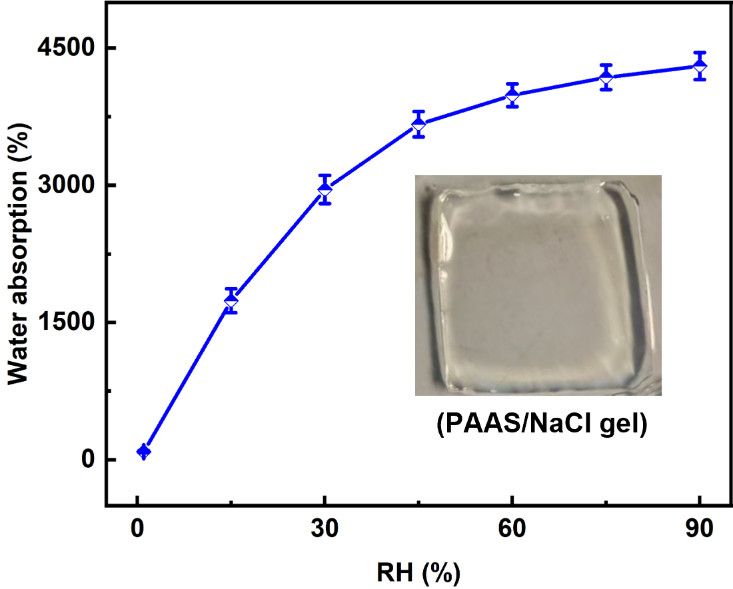


Figure S7. The water absorption and photo of PAAS/NaCl at different RH. The PAAS/NaCl gels were stored in the environments with different RH for 24 h and then were recorded with water absorption.


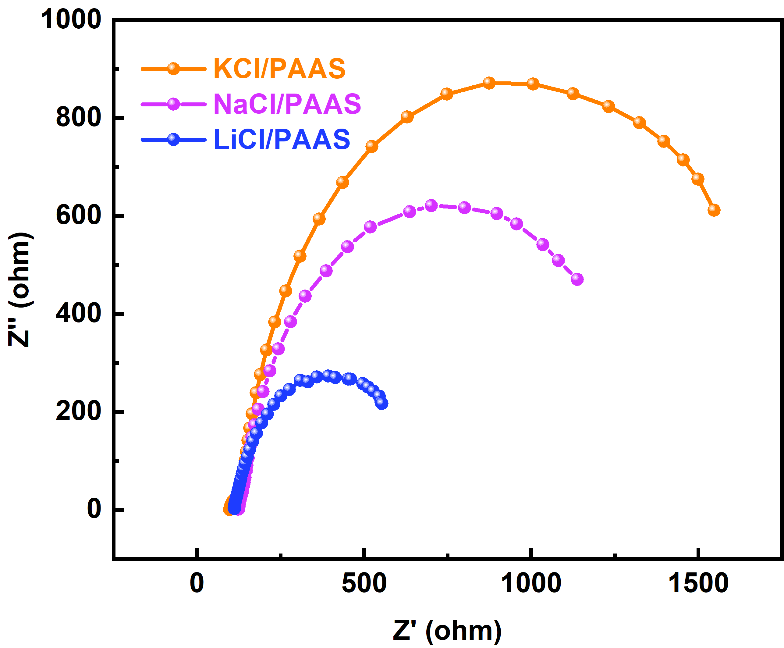


Figure S8. EIS of hygroelectric generators with different polymer systems at room humidity of 55%. The 3 wt.% PAAS and 1.2% salts were dispersed in D.I. water in the fabrication of different hygroelectric generators.


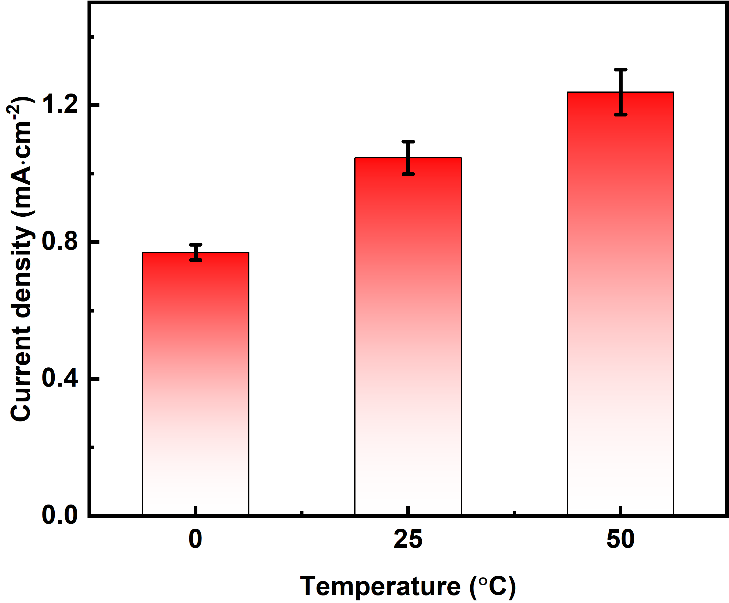


Figure S9. The current density of hygroelectric generators at different operation temperatures with 55% RH. The hygroelectric generators were stored at different temperatures for 24 h before recording the current density.


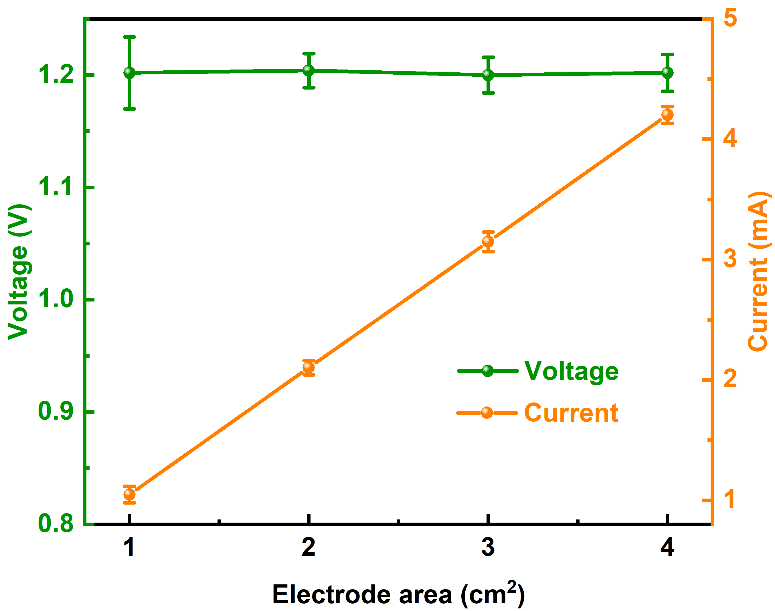


Figure S10. Electric outputs of the hygroelectric generator unit with different electrode areas at room humidity of 55%. The area ratio of electrode and functional layer keeps the same for hygroelectric generators with different electrode areas.


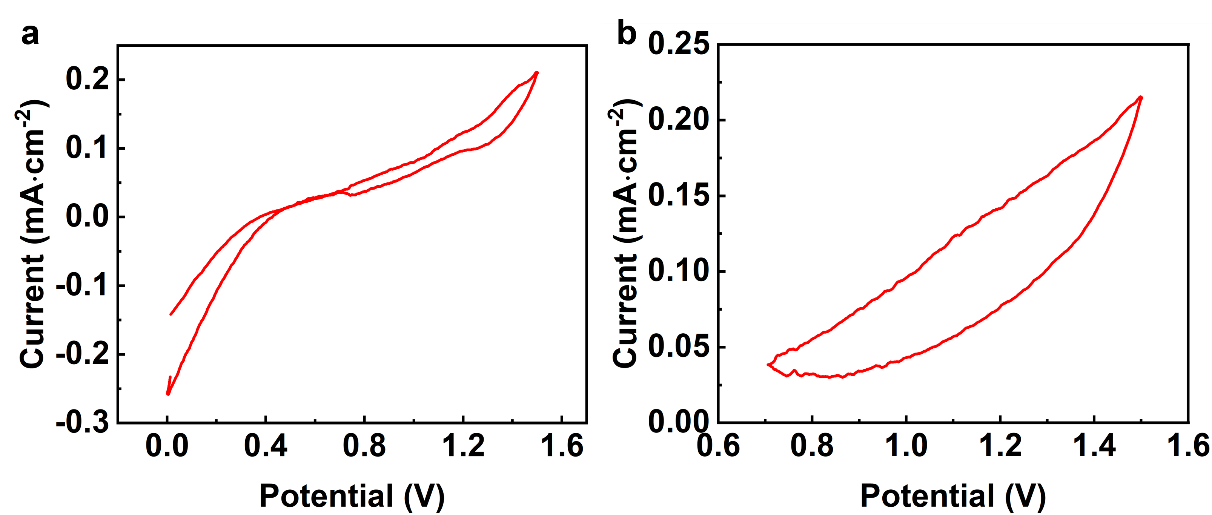


Figure S11. Cyclic voltammetry curve of a hygroelectric generator unit with Al and graphene as electrodes at 90% RH with a scan rate is 0.5 mV·s^-1^. a) Cyclic voltammetry curve of devices at 0–1.5 V. b) Cyclic voltammetry curve of devices at 0.7–1.5 V.


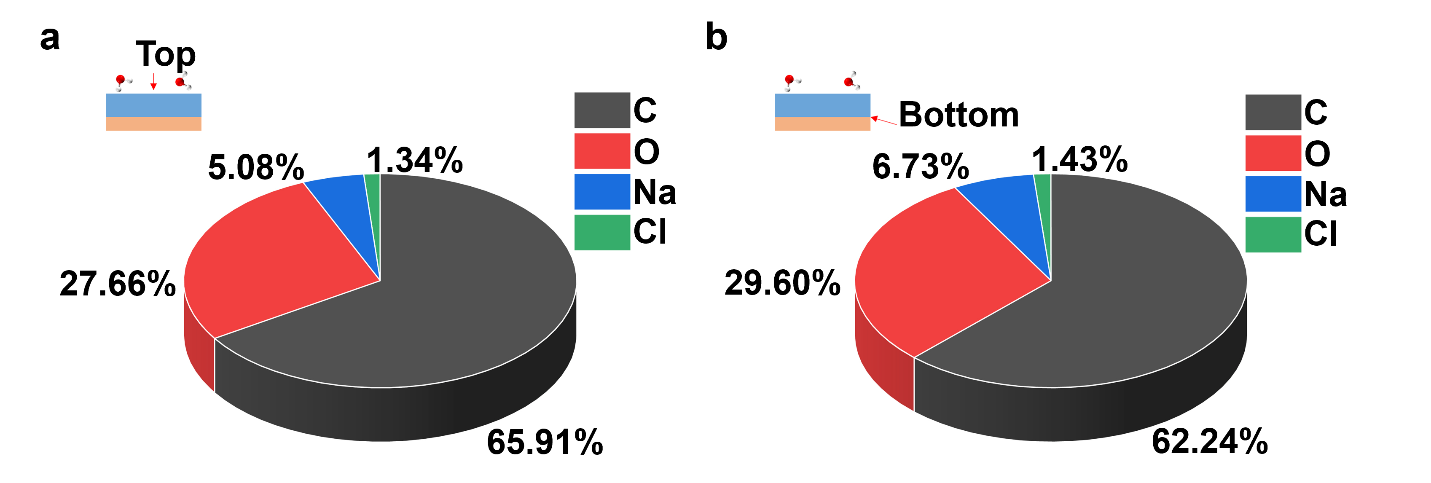


Figure S12. Element distribution from X-ray photoelectron spectroscopy of PAAS/NaCl layer after operation with Al electrode at 90% RH for 2 weeks. a) X-ray photoelectron spectroscopy on the top surface. a) X-ray photoelectron spectroscopy on the bottom surface. The bottom surface is tested after peeling gel off the substrate.


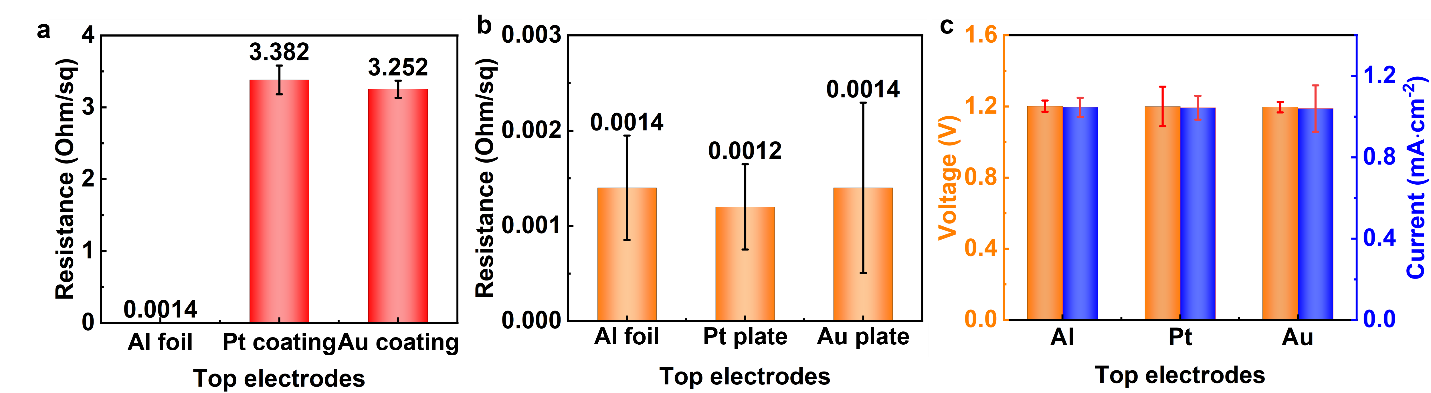


Figure S13. Electric performance of devices with different electrodes. a) Electric resistance of Al foil, Pt coating and Au coating. Pt coating and Au coating were fabricated by sputtering Pt or Au on the PET substrates. b) Electric resistance of commercial Al foil, Pt plate and Au plate. c) Electric outputs of devices with Al foil, Pt plate or Au plate as top electrodes.


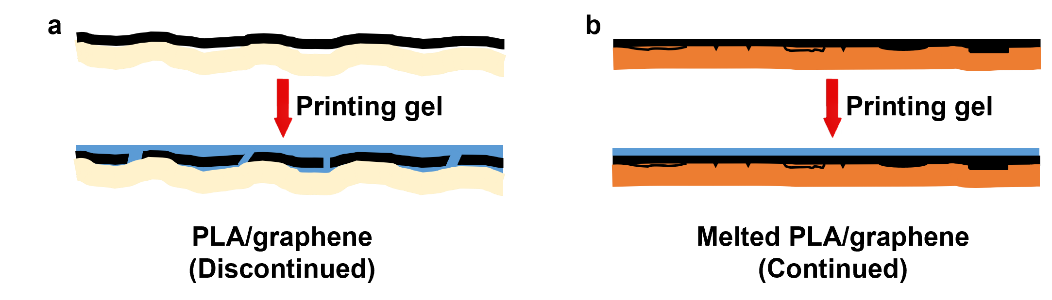


Figure S14. Schematical illustration of different PLA/graphene structures after printing PAAS/NaCl gels onto PLA/graphene. a) PLA/graphene without hot-pressing. b) PLA/graphene with hot-pressing.


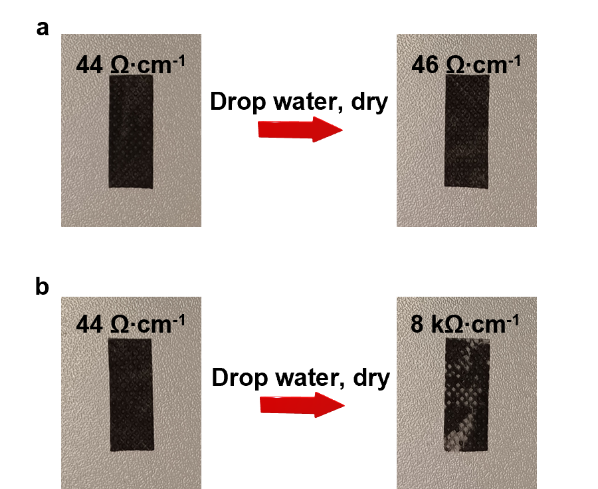


Figure S15. Photo of graphene electrode on PLA fabric with a size of 1.0×2.5 cm^2^ after dropping water and drying at 50 °C for 5 h. a) Graphene electrode on PLA fabric with hot-pressing. b) Graphene electrode on PLA fabric without hot-pressing.


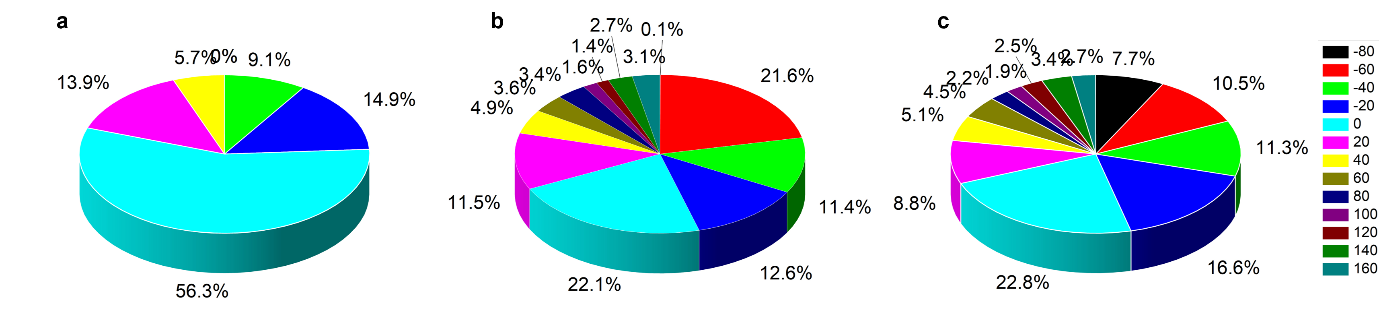


Figure S16. The area percentage in each electrostatic potential range of a) PVA, b) PAAS and c) PAAS/NaCl.


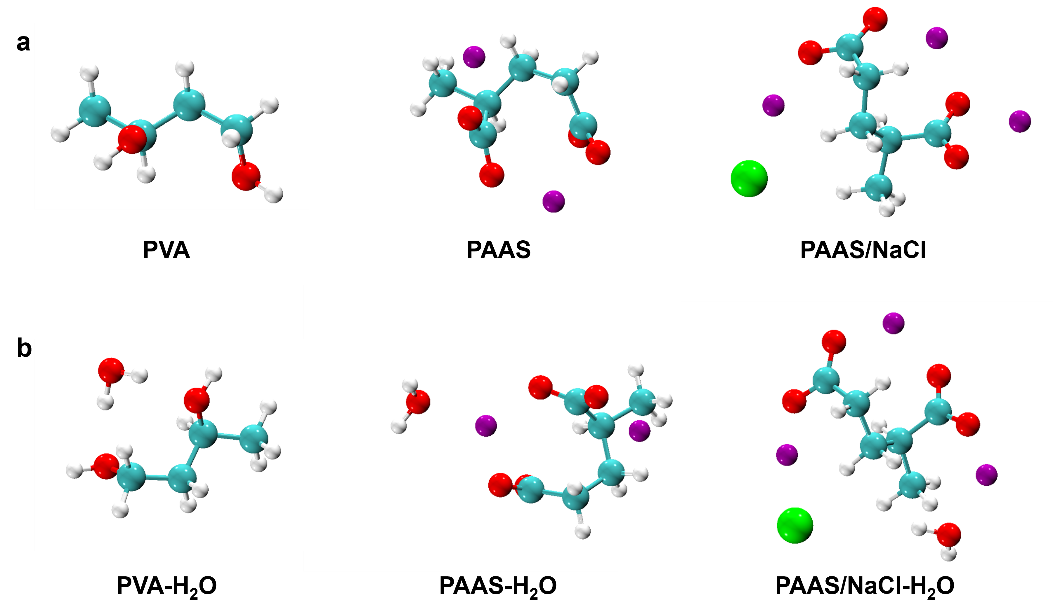


Figure S17. Molecular structures of different polymer systems from DFT calculations. a) Polymers. b) Polymers with water molecules.


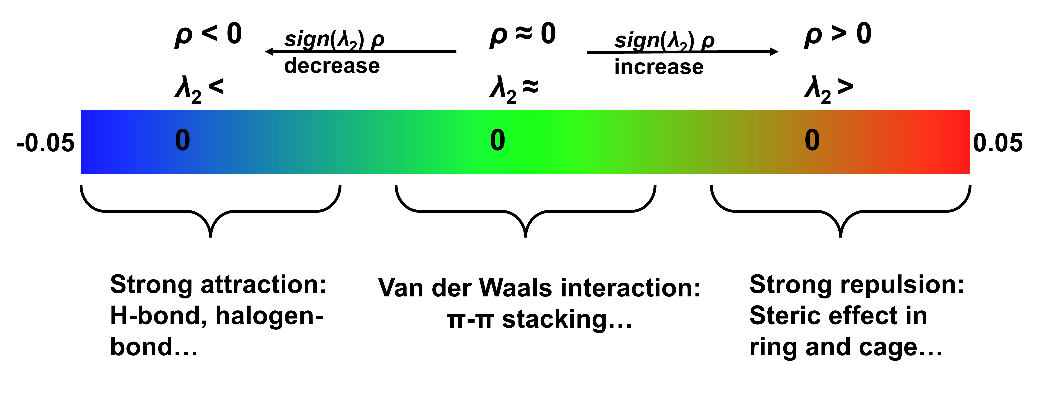


Figure S18. Isosurface map of interactive region between polymers and H_2_O with sign(*λ*_2_)*ρ* mapped on the isosurface. Blue and red colors represent attraction and repulsion, respectively.


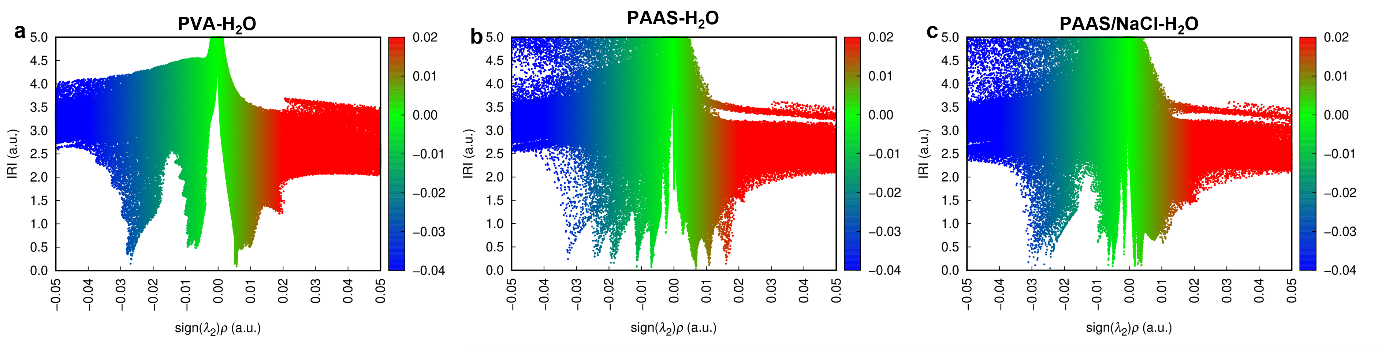


Figure S19. The scatter plots of the IRI versus the electron density multiplied by the sign of the second Hessian Eigen value (λ_2_) for a) PVA-H_2_O, b) PAAS-H_2_O, c) PAAS/NaCl-H_2_O.


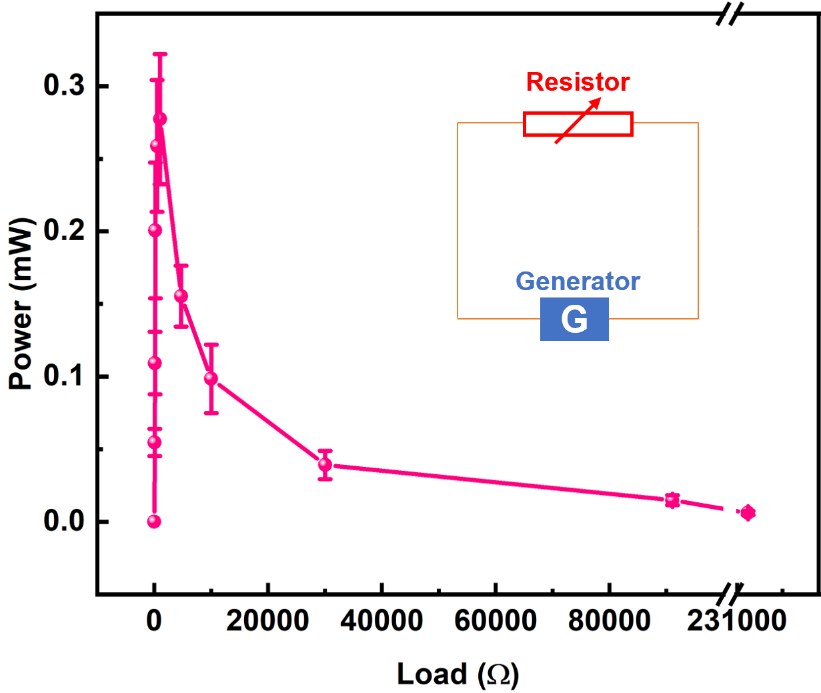


Figure S20. Power output of the external resistors with different resistance at room humidity of 55%. Power outputs are calculated with voltage and current outputs of external resistors connected to the hygroelectric generator unit.


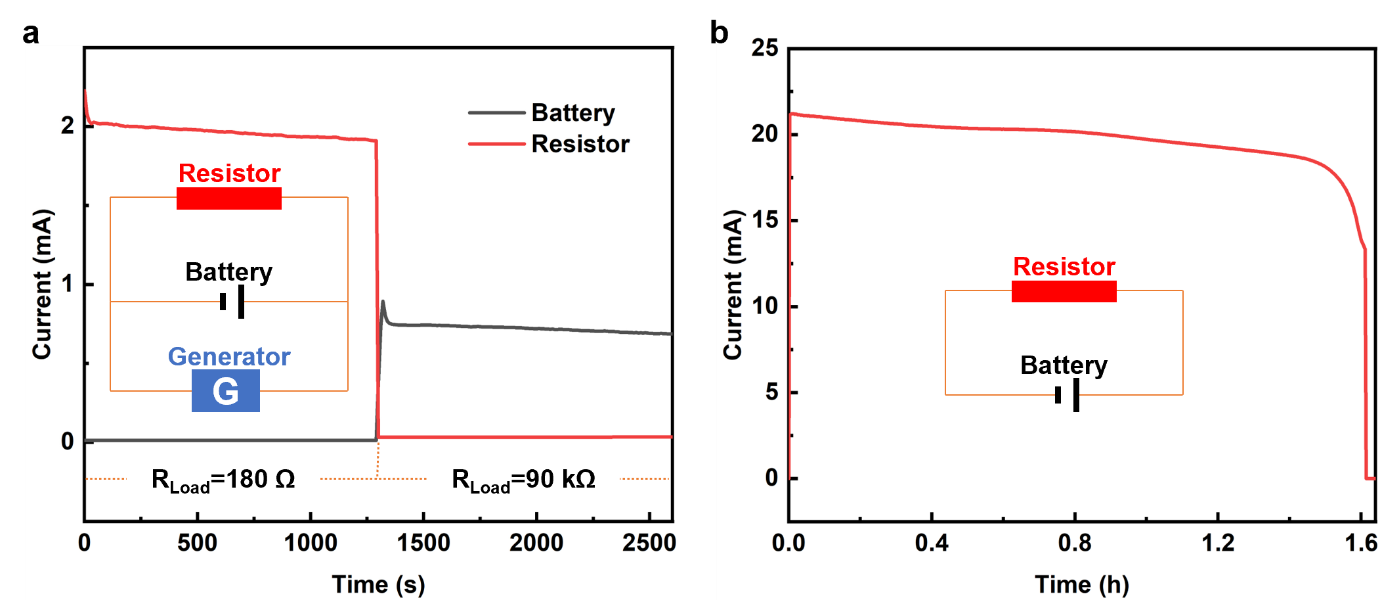


Figure S21. Charging and discharging of power supply system. a) The current outputs of a commercial Li-battery and external resistors with different resistance charging by 3 hygroelectric generator units connected in series. b) Discharge current of battery connected to a load with 180 Ω.


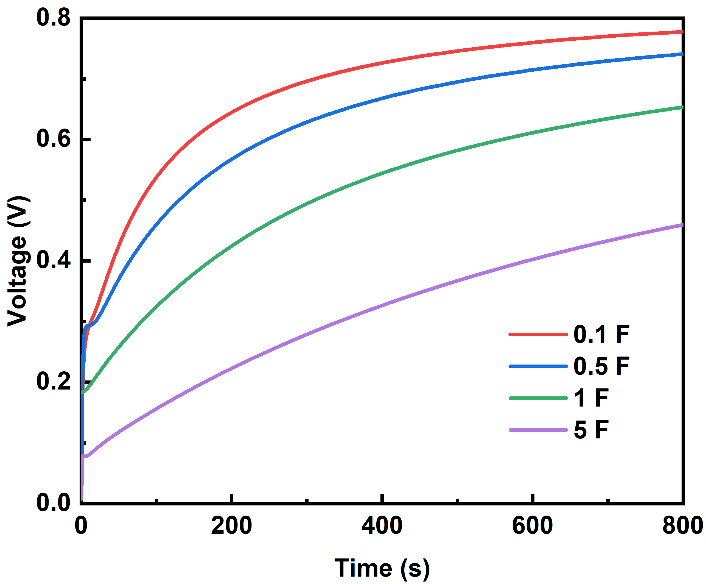


Figure S22. The voltages of capacitors with different capacities, charged by the single hygroelectric generator unit at room humidity of 55%.


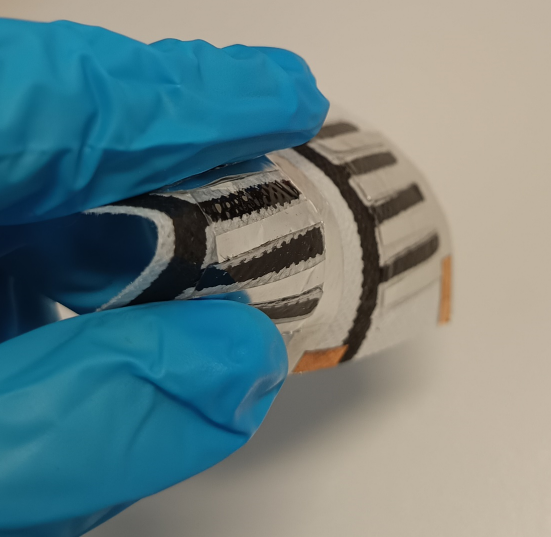


Figure S23. Photo of the bended hygroelectric generator arrays with 2 hygroelectric generator units in series and 4 hygroelectric generator units in parallel. The Cu tape was used to connect hygroelectric generator units in series.


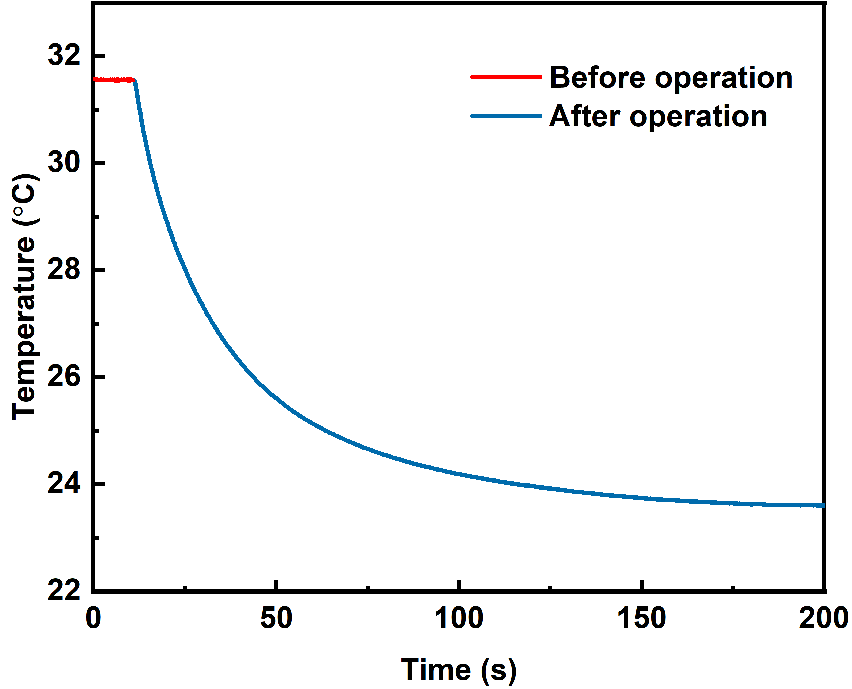


Figure S24. Temperature of cooling skin surface by electric fan. The temperature signals were delivered by wireless sensor powered by generators.

**References:**

[1] X. M. Liu, H. Y. Gao, J. E. Ward, X. R. Liu, B. Yin, T. D. Fu, J. H. Chen, D. R. Lovley, J. Yao, *Nature* 2020, 578, 550.

[2] Y. X. Huang, H. H. Cheng, C. Yang, P. P. Zhang, Q. H. Liao, H. Z. Yao, G. Q. Shi, L. T. Qu, *Nat. Commun.* 2018, 9, 4166.

[3] Y. X. Huang, H. H. Cheng, C. Yang, H. Z. Yao, C. Li, L. T. Qu, *Energy Environ. Sci.* 2019, 12, 1848.

[4] H. H. Cheng, Y. X. Huang, F. Zhao, C. Yang, P. P. Zhang, L. Jiang, G. Q. Shi, L. T. Qu, *Energy Environ. Sci.* 2018, 11, 2839.

[5] R. B. Zhu, Y. Z. Zhu, L. Hu, P. Y. Guan, D. W. Su, S. Zhang, C. Liu, Z. H. Feng, G. Y. Hu, F. D. Chen, T. Wan, X. W. Guan, T. Wu, R. Joshi, M. Y. Li, C. Cazorla, Y. R. Lu, Z. J. Han, H. L. Xu, D. W. Chu, *Energy Environ. Sci.* 2023, 16, 2338.

[6] D. Z. Shen, M. Xiao, G. S. Zou, L. Liu, W. W. Duley, Y. N. Zhou, *Adv. Mater.* 2018, 30, 1705925.

[7] X. Gao, T. Xu, C. X. Shao, Y. Y. Han, B. Lu, Z. P. Zhang, L. T. Qu, *J. Mater. Chem. A.* 2019, 7, 20574.

[8] W. Q. Yang, X. K. Li, X. Han, W. H. Zhang, Z. B. Wang, X. M. Ma, M. J. Li, C. X. Li, *Nano Energy* 2020, 71, 104610.

[9] Y. H. Hu, W. F. Yang, W. Wei, Z. Q. Sun, B. Wu, K. R. Li, Y. G. Li, Q. H. Zhang, R. Xiao, C. Y. Hou, H. Z. Wang, *Sci. Adv.* 2024, 10, eadk4620.

[10] Y. X. Huang, K. Zhou, H. H. Cheng, T. C. He, H. Y. Wang, J. X. Bai, C. Yang, T. L. Guang, H. Z. Yao, F. Li, G. J. Hou, Z. P. Xu, L. T. Qu, *Adv. Funct. Mater.* 2024, 34, 2308620.

[11] J. C. Liang, Y. D. Wang, X. K. Ma, X. M. Song, H. Z. Wang, T. Y. Shen, J. J. Sun, Y. Hu, Y. Z. Liu, Z. A. Wu, T. C. Yu, Z. X. Tie, Z. Jin, *Adv. Funct. Mater.* 2024, 2418834.

[12] D. Z. Shen, F. Z. Li, J. Zhao, R. Wang, B. Li, Z. C. Han, L. L. Guo, P. C. Han, D. Q. Yang, H. H. Kim, Y. J. Su, Z. X. Gong, L. M. Zhu, *Adv. Sci.* 2024, 11, 2408954.

[13] G. T. Zan, W. Jiang, H. Y. Kim, K. Y. Zhao, S. Y. Li, K. Lee, J. Jang, G. Kim, E. A. Shin, W. Kim, J. W. Oh, Y. Kim, J. W. Park, T. Kim, S. Lee, J. H. Oh, J. Shin, H. J. Kim, C. Park, *Nat. Commun.* 2024, 15, 10056.

[14] D. Maity, M. Fussenegger, *Adv. Sci.* 2023, 10, 2300750.

[15] Y. H. Liu, Z. H. Li, X. J. Yang, Y. Yang, X. S. Li, Y. Jiang, Y. Gao, L. Y. Wang, W. Lü, *Adv. Funct. Mater.* 2024, 34, 2407204.
